# Large- and Small-Scale Beam-Steering Phased Array Antennas Using Variable Phase BLC for Millimeter-Wave Applications

**DOI:** 10.3390/s25123714

**Published:** 2025-06-13

**Authors:** Fayyadh H. Ahmed, Salam K. Khamas

**Affiliations:** Electromagnetics, Wireless Hardware & RF Devices Group, School of Electronic and Electrical Engineering, University of Sheffield, Sheffield S1 3JD, UK; s.khamas@sheffield.ac.uk

**Keywords:** BLC, millimeter-wave communication, phase array antenna, switchable phase shift

## Abstract

This paper presents a novel switchable branch-line coupler (BLC) designed to achieve variable phase shifts while maintaining a constant output power. The proposed design incorporates low stepwise phase shifters with incremental phase shifts of 10° to 20°, covering phase ranges from −3° to 150°. The initial structure is based on a 3 dB branch-line coupler with arm electrical lengths of 3λ_g_/2. A novel delay line structure is integrated within the BLC arms, consisting of a λ_g_/4 section bridged by a tapered stripline to accommodate a PIN diode switch, thereby altering the current path direction. Additionally, two interdigital capacitors (IDCs), uniquely mounted on a crescent-shaped extension, are implemented alongside the tapered line to elongate the current path when the PIN diode is in the OFF state. By controlling the PIN diode states, the delay time is differentially adjusted, resulting in variable differential phase shifts at the output ports. To validate the functionality, the proposed BLC was integrated with a two-element antenna array to demonstrate differential beam steering. The measurement results confirm that the phased array antenna can switch its main beam between −27° and 25° in the elevation plane, achieving an average realized gain of approximately 7 dBi. The BLC was designed and simulated using CST Microwave Studio and was fabricated on an RO4003C Roger substrate (ε_r_ = 3.55, 0.406 mm). The proposed design is well-suited for future Butler matrix-based beamforming networks in antenna array systems, particularly for 5G wireless applications.

## 1. Introduction

The development of beam-reconfigurable antennas has garnered increased attention in recent years, as they represent a critical requirement for advanced communication systems, such as 5G and beyond. Common techniques for beamforming include digital beamforming [1,2], phase-shifting techniques [3,4], and analog beamforming networks (BFNs) [5,6,7]. Among these, Butler matrices are widely recognized as efficient, passive, switched-beam networks due to their simplicity, low cost, and ease of implementation. A Butler matrix generates specific amplitude and phase distributions at its output ports, which, when combined with an antenna array, enables the formation of discrete beam directions.

A standard Butler matrix generally consists of couplers, phase shifters, and crossovers. Among these, the coupler is a key component that significantly impacts both the physical and electrical properties of the matrix. Conventional hybrid couplers are designed to produce a fixed, predefined phase difference, maintaining a steady 90° phase shift when driven by signals from various input ports. However, beam-steering arrays made from conventional components lack the spatial resolution required by modern communication systems to meet the flexibility needs of emerging wireless networks, so incorporating switchable components has become necessary.

The ability to reconfigure individual components within a system is increasingly important, not only for established mature systems but also for application-specific ones [1]. This reconfigurability, driven by the need for flexible advanced operations, also helps reduce implementation costs. Reconfigurable components enable performance optimization tailored to specific operational conditions, making them essential for achieving the flexibility required in emerging wireless communication systems.

In beamforming, phase control is a critical factor in enhancing system performance. For instance, accurately managing the phase of signals received by each antenna element is essential for effective beamforming. As a result, phase reconfiguration has become a key feature for hybrid couplers in current and next-generation wireless communication systems. However, achieving phase reconfigurability within a coupler is more complex than within a phase shifter, as it requires the simultaneous adjustment of multiple circuit parameters to meet essential criteria, such as the expected return loss, equal power division, and the desired phase characteristics. In the literature, various structures have been reported to enable phase reconfiguration and tunability. For example, incorporating varactor diodes with defected ground structures (DGSs) enables a broad phase shift range, as demonstrated in [8]. The varactor diodes are mounted on the DGSs within the termination loads to precisely control the substantial phase variation in the DGS at resonance. In addition, four tunable phase shifters were connected to a 4 × 4 Butler matrix to provide a 360° phased array feeding network [9]. Similar approaches have been explored in other studies [10,11,12]. However, this technique has several limitations, including a narrow bandwidth determined by that of the phase shifter. Additionally, the insertion loss (IL) is relatively high. Various tunable phase shifter topologies have been proposed in the literature [13,14]. Nonetheless, integrating these designs into a Butler matrix remains challenging due to factors such as bulkiness, high insertion loss, and increased complexity.

Other researchers adopted a reflection-type phase shifter technique [15], which involves the integration of an impedance-transforming quadrature coupler (QC) with series-resonated varactors to achieve equalized phase shifting. The impedance-transforming quadrature coupler is used to enhance the maximum achievable relative phase shift for a given varactor, even within a limited capacitance range. However, this approach encounters several challenges, including substantial insertion loss variation in the phase shifter due to the parasitic effects of the varactors during phase tuning. Additionally, this configuration is unsuitable to be used as a unit cell in a Butler matrix system, as two ports of the branch-line coupler or QC (typically the output ports in a conventional BLC) are loaded with varactor diodes, which impairs functionality. Furthermore, an electrically tunable-length hybrid coupler was reported on in [16,17,18,19]. The tunability of the BLC length was achieved using varactor diodes positioned across the coupler branches. By adjusting the bias voltage applied to the varactor diodes, the effective length of the BLC transmission line can be reconfigured, thereby modifying the phase across the output line. However, incorporating varactor diodes into the BLC (or hybrid coupler) transmission line introduces significant phase errors, in addition to limited power handling capability and increased losses due to parasitic elements, which become more severe at millimeter-wave frequencies [20].

Recently, several new techniques have been adopted to alter the phase shift in phased array antenna systems. One such method is voltage-controlled phase shifting using liquid crystals (LCs), which has been implemented in antenna arrays [21]. In this approach, each element features a meandered transmission line over an LC layer. A bias voltage modulates the permittivity of the LC, enabling a beam steering of up to ±45°. However, the multilayer substrate—including the grooved LC containment—and the use of 25 four-channel power dividers for 100 elements significantly increase both fabrication and system complexity. The phase-controlled synthesis of dual-mode radiators (patch and loop) is demonstrated in [22], where each array element includes two feed ports—one for broadside patch excitation and another for conical loop radiation. Beam steering is achieved by varying the phase difference between the two feeds. However, the approach supports only a few discrete phase states, and the dual-port design doubles the number of connectors, significantly increasing feeding network complexity. A physical beam redirection technique using a metasurface lens and a defocused feed array (DAA) was proposed in [23]. In this approach, a fixed metasurface lens reshapes the wavefronts from the DAA, and beam steering is achieved by selectively exciting different sub-arrays. While this method eliminates the need for complex amplitude or phase control networks, it supports only discrete scanning, limited to approximately ±15°–16°.

Materials that alter their properties under electromagnetic exposure are increasingly vital in phase shifter design, where intrinsic tunability can eliminate the need for additional components. Barium hexaferrite, with low microwave loss, high resistivity, and strong magnetic anisotropy, is well-suited for high-frequency applications [24]. Its ferrimagnetic nature allows for RF phase control via ferromagnetic resonance (FMR), enabling non-mechanical phase shifting when embedded in waveguides and tuned by an external magnetic field. Similarly, defect-engineered perovskite manganites offer tunable magneto-resistive and magneto-capacitive properties with strong dielectric and magnetic responses at high frequencies [25]. Their low dielectric loss and thermal stability make them ideal for mmWave circuits in 5G systems, particularly in components like Butler matrices and beamforming networks.

This paper introduces a novel differential phase reconfigurability method by adjusting the length of the BLC transmission line based on a defective microstrip line (DML) concept. This approach allows the electrical length of the BLC transmission line to be modified without altering its physical length, eliminating the need for additional components like standalone phase shifters or varactor diodes commonly used in prior studies. The concept is based on extending the electrical length of the current flowing from the input to the output by introducing a specific deflection in the arms of the BLC. Additionally, a PIN switch is used to control the current path, where the PIN diode is integrated into the transmission line rather than being a separate standalone unit.

The design concept and circuit model, along with a detailed analysis of the variable phase BLC, are presented in Section 2 to illustrate the working principle of the proposed design. To verify the design feasibility in generating a variable phase shift at both large and small scales, a prototype was fabricated. The measured parameters were then compared with the simulation results in Section 3, demonstrating strong agreement. Finally, two conventional microstrip antennas were incorporated to assess the proposed BLC’s suitability for Butler matrix circuits and its capability for gradual beam steering. The radiation beam was measured for multiple diode switch states, as presented in Section 4.

## 2. Design and Analysis

### 2.1. Variable Phase Transmission Line Module

**Figure 1:**
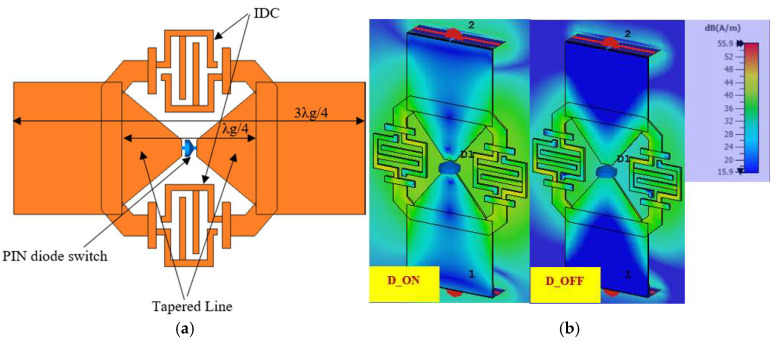
(**a**) The proposed variable delay line, (**b**) current distribution visualization in different diode states.

The transmission line unit used in constructing the branch-line coupler is shown in Figure 1a. The length of the transmission line is set to 3λ/4 instead of the conventional λ_g_/4. This decision is driven by the requirement to design a coupler operating at millimeter-wave frequencies, where a quarter-wavelength transmission line would result in an extremely compact structure, potentially restricting the feasibility of subsequent modifications. Consequently, a length of three-quarters of a guided wavelength (3λ_g_/4) is chosen to allow for greater flexibility in adjustments. To incorporate the proposed novel reconfigurable delay structure, a λ_g_/4 section is removed from the center of the 3λ_g_/4 transmission line. This structure consists of two tapered transmission lines positioned opposite each other, separated by a square slot measuring 0.2 mm × 0.2 mm that houses a PIN diode switch. Alongside the tapered lines, a crescent-shaped extension is incorporated to accommodate a novel interdigital capacitor (IDC), ensuring RF continuity when the diode is in the OFF state. The crescent-shaped arms with the IDC occupy a minimal additional surface area, as they are accommodated within the cleared space created by the tapered transmission lines.

**Figure 2:**
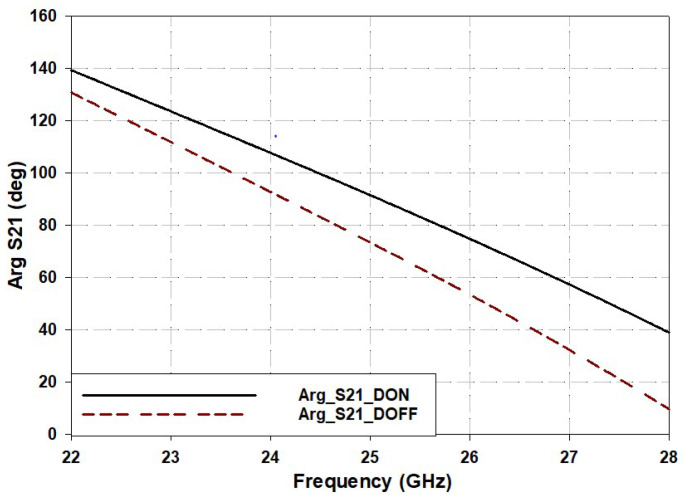
The S phase delay of the proposed variable phase delay transmission line.

The delay line structure operates in two distinct modes depending on the state of the PIN diode. When the diode is “ON”, the current mainly flows through the central region of the delay line via a tapered line before passing through the diode, as depicted in Figure 1b. This configuration approximates the behavior of a conventional transmission line. Due to the shorter path traversed by the current, the electrical length remains finite, resulting in a specific phase difference at the output port, as demonstrated in Figure 2.

Conversely, when the PIN diode is “OFF”, the central path is interrupted, causing the current to accumulate at the center, as shown in Figure 1b, before being redirected along a longer outer route through the IDC structure. This extended electrical path results in a greater phase delay at the output port compared to the “ON” state. Thus, by toggling the PIN diode between the “ON” and “OFF” states, it is possible to achieve different phase differences at the output port without altering the physical length of the transmission line.

**Figure 3:**
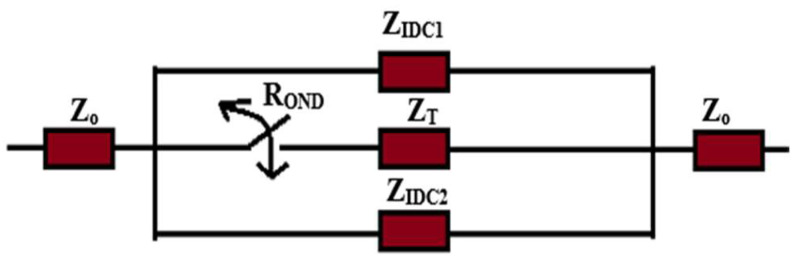
Lumped impedance model of proposed variable phase structure.

Figure 3 presents the lumped impedance model of the variable phase shifter structure, illustrating three equivalent parallel impedances. The outer impedances correspond to that of the IDC circuit, while the central impedance represents that of the middle tapered line connected in series with the forward resistance of the PIN diode. For effective impedance matching, the combination of parallel impedances must closely approximate the characteristic impedance of the transmission line to which they are connected, as outlined below.

(1) $$Z_{sh}\approx Z_{0}=\frac{1}{Z_{IDC1}}+\frac{1}{Z_{IDC2}}+\frac{1}{Z_{T}+R_{OND}}$$

where Z_sh_ and Z_IDC1_ and Z_IDC2_ represent the total equivalent shunt impedance and the input impedances of IDCs 1 and 2 in the variable phase shifter, respectively, while Z_T_ and R_OND_ denote the impedance of the tapered line and the forward resistance of the PIN diode, respectively. However, in the “OFF” state, the central impedance is effectively removed from the circuit due to the diode’s infinite resistance. Consequently, the impedances of the capacitors must be selected with extreme precision to ensure that the equivalent impedance of the circuit in both diode states is close enough to the characteristic impedance of the connected transmission line in both the ON and OFF states.

**Figure 4:**
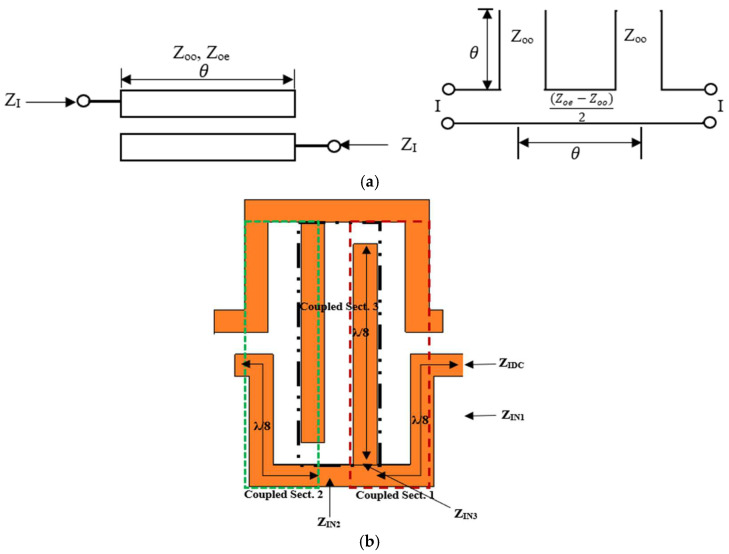
(**a)** Schematic of open-end coupled line and its equivalent circuit, (**b**) proposed IDC structure.

The inherent electromagnetic coupling between parallel transmission lines can be effectively leveraged in the design of various microwave components, including filters, directional couplers, and IDCs. However, to determine the IDC input impedance, the impedance characteristics of the open-stub coupled lines will be analyzed [26]. A symmetrical open-end coupled line configuration can be represented by its equivalent open-wire transmission line circuit, as shown in Figure 4a. The input image impedance for this arrangement is given by [27].

(2) $$Z_{I1}=\frac{{\left[{\left(Z_{oe}-Z_{oo}\right)}^{2}-{\left(Z_{oe}-Z_{oo}\right)}^{2}{cos}^{2}\theta \right]}^{1/2}}{2sin\theta }$$

where Z_oe_ and Z_oo_ represent the even and odd characteristic impedances of the coupled line, respectively, and θ denotes the electrical length of the coupled line.

By incorporating the concept illustrated in Figure 4a into the proposed IDC design, the structure can be segmented into three sections (or pairs) of λ_g_/8 coupled lines, as depicted in Figure 4b. However, examining the structure from its input port, it can be deduced that the total input impedance Z_IDC_ is equivalent to their parallel combination as follows:

(3) $$\frac{1}{Z_{IDC}}=\frac{1}{Z_{IN1}}+\frac{1}{Z_{IN2}}+\frac{1}{Z_{IN3}}$$

where Z_IN1_, Z_IN2_, and Z_IN3_ are the impedances resulting from coupled sections 1, 2, and 3, respectively. By precisely selecting the dimensions and spacing between the coupled lines of the IDC, a transmission line with favorable return and insertion loss characteristics, as well as varying phase differences, can be realized. This approach eliminates the need to alter the physical length or incorporate parallel surface-mounted capacitors into the transmission line. Such a transmission line can be effectively employed in a hybrid coupler to achieve a variable (or rather differential) phase shift.

To evaluate the transmission loss across the IDC, a conventional coupler method was adopted in [28], where the coupled line is modeled as a four-port conventional coupler. The isolated and transmission ports are assumed to be terminated with an open circuit, while the coupled ports are terminated with the characteristic impedance Z_o_. Under these conditions, the transmission loss between the coupled ports can be expressed as follows:

(4) $$T=20{log}_{10}{\left({1-\left(\frac{\left|Z_{in}-Z_{0}\right|}{\left|Z_{in}+Z_{0}\right|}\right)}^{2}\right)}^{1/2}$$

Therefore, in the proposed structure of Figure 4b, the transmission loss across the proposed IDC can be determined by first calculating the input impedance of the structure using (3) and then applying the following formula.

(5) $$T=20{log}_{10}{\left({1-\left(\frac{\left|Z_{IDC}-Z_{0}\right|}{\left|Z_{IDC}+Z_{0}\right|}\right)}^{2}\right)}^{1/2}$$

**Figure 5:**
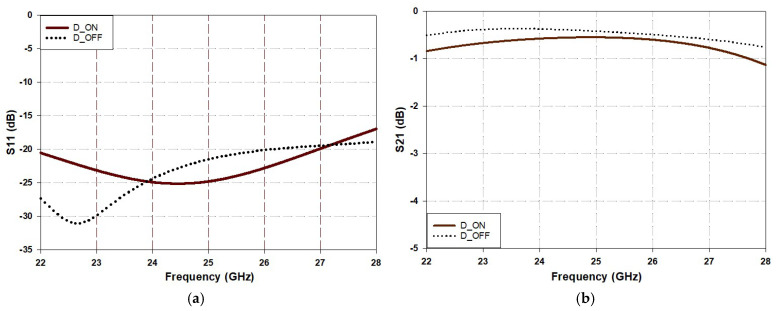
Scattering parameters of proposed IDC in Figure 4b: (**a**) S_11_ and (**b**) S_21_.

Figure 5 illustrates the simulated S_11_ and S_21_ of the proposed variable phase shift delay line. As expected, the return loss slightly shifts downward in the OFF state because the current follows a slightly longer path compared to the ON state. Additionally, in the ON state, S_21_ is observed to be lower than that in the OFF state. This is due to the losses associated with the forward ON resistance. In both cases, although there is a slight shift in the resonant frequency between the different diode states, the transmission line remains matched. Furthermore, the transmission coefficient is not significantly affected and maintains its ability to transmit signals with minimal losses.

### 2.2. BLC Circuit Implementation and Evaluation

A hybrid BLC is a critical component for efficiently splitting an input signal into two equal outputs with a precise 90° phase difference. This characteristic makes it particularly suitable for use in phased array feeding networks for 5G applications. The scattering (S) matrix of a 90° hybrid coupler, also referred to as a quadrature hybrid, characterizes its performance in terms of power division, phase shift, and port isolation. For an ideal, lossless, and perfectly matched 4-port 90° hybrid coupler, the S-matrix is expressed as follows [29]:

(6) $$S=\frac{1}{\sqrt{2}}\left[\begin{array}{cccc} 0 & 1 & 0 & j\\ 1 & 0 & j & 0\\ 0 & j & 0 & 1\\ j & 0 & 1 & 0 \end{array}\right]$$

The S-parameter matrix describes the system’s signal transmission and reflection behavior. Ideal performance requires |S_11_|, |S_22_|, |S_33_|, and |S_44_| to be zero and |S_21_|, |S_12_|, |S_32_|, |S_23_|, |S_14_|, |S_41_|, |S_43_|, and |S_34_| to equal ($1/\sqrt{2}$). Isolation demands that |S_31_|, |S_12_|, |S_24_|, and |S_42_| be zero, while a 90° phase difference between output ports 2 and 4 is necessary, independent of amplitude. This phase shift is governed by the coupler’s geometry and electrical length. In the proposed design, a differential phase shift is achieved by introducing a variation in the electrical length of the BLC, represented by a differential length ∆l, rather than maintaining the constant 90° phase shift typical of conventional designs. Under this configuration, the S-matrix is formulated as follows:

(7) $$S=\frac{1}{\sqrt{2}}\left[\begin{array}{cccc} 0 & 1 & 0 & e^{\emptyset \mp ∆\theta }\\ 1 & 0 & e^{\emptyset \mp ∆\theta } & 0\\ 0 & e^{\emptyset \mp ∆\theta } & 0 & 1\\ e^{\emptyset \mp ∆\theta } & 0 & 1 & 0 \end{array}\right]$$

where ∅ is a 90° phase shift due to the standard BLC circuit, and $∆\theta =\frac{2\pi ∆l}{\lambda }$ is the differential phase due to the differential length ∆l.

**Figure 6:**
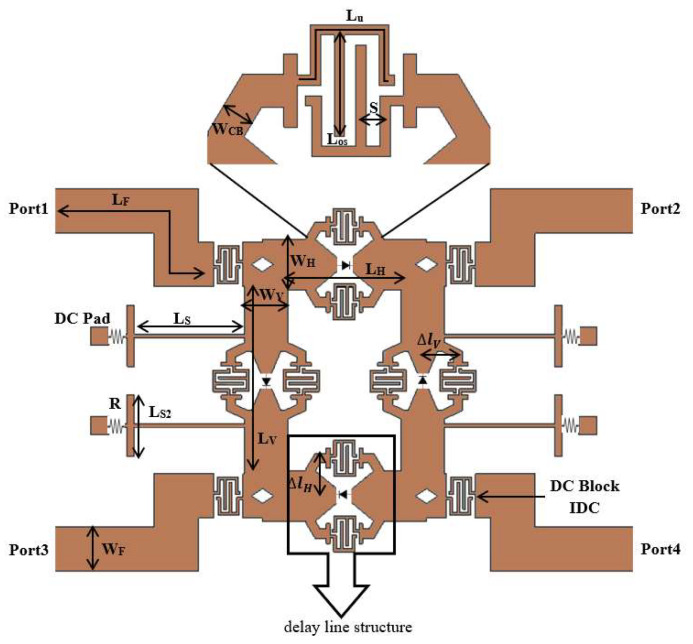
Schematic representation of proposed variable phase shift coupler.

The proposed variable phase shift BLC is illustrated in Figure 6, with the corresponding parameter values presented in Table 1. These parameters were optimized using CST Microwave Studio 2023. The optimization objectives included achieving a range of differential phase shifts while maintaining acceptable insertion and return losses for various input ports. Additionally, the design aimed to maintain structural simplicity, eliminating the need for surface-mount devices (SMDs) for diode DC biasing.

The proposed structure consists of four transmission lines, with a pair of horizontally placed lines of length 3λ_g_/4 (L_H_) and characteristic impedance $Z_{0}/\sqrt{2}$, intersecting with a vertically oriented pair of lines (L_V_) with characteristic impedance Z_0_. The widths of these lines were determined using [30] based on the Roger RO4003C substrate parameters of ε_r_ = 3.55 and a height of 0.406 mm. Each transmission line incorporates a delay line structure, which includes a tapered line at its center, flanked by a pair of IDC structures around the tapered line. At the core of the structure lies a slot with dimensions of 0.2 × 0.2 mm to accommodate a PIN diode, allowing the current path to alternate between the central section and the lateral IDC sections. This mechanism effectively alters the electrical length of the line. It is worth noting that the dimensions of the delay line structures associated with the horizontal lines differ from those connected to the vertical lines. This variation is designed to maximize the diversity in electrical lengths, thereby achieving a broader range of differential phase shifts.

**Figure 7:**
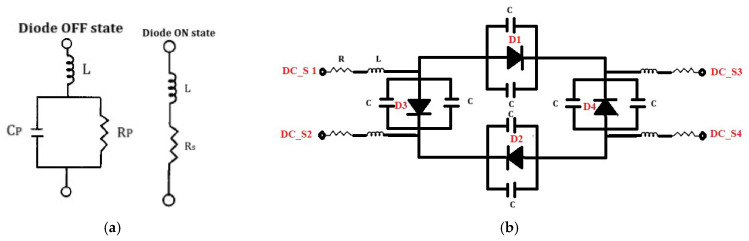
(**a**) Equivalent circuit of PIN diode in its ON and OFF states, (**b**) DC biasing circuit for diode configuration shown in Figure 6.

In the simulations, the PIN diode (MA4AGFCP910) was represented using an equivalent circuit model, as shown in Figure 7a. In the ON state, this diode was modeled as a series combination of a resistance (RS) of 5.2 Ω and an inductance (LS) of 0.5 nH. Conversely, in the OFF state, the diode was modeled as a parallel combination of a resistance (RP) of 300 kΩ and a capacitance (CP) of 0.021 pF, based on the specifications provided in the product datasheet [31]. The feeding lines of the coupler (LF) are 3λ_g_/2 in length, and at the end of each line on the coupler side, there is a DC block (IDC) to prevent the leakage of the DC current required for diode switching into the RF supply. To further elucidate the working principle of the diode combination in its biasing circuit, the DC biasing configuration is illustrated in Figure 7b. In this circuit, the supply is applied at the DC ports, labeled as DC_S1 through DC_S4. The DC supply operates at three voltage levels of 2.5 V, 0 V, and −2.5 V depending on the biasing requirements of specific diodes. The details of the various diode configurations, along with the corresponding applied DC voltages, are presented in Table 2.

A variety of differential phases can be generated simply by switching the four diodes located on the four arms. This is achieved by applying DC voltage to each diode, thereby altering the path of surface currents traveling from the input to the output ports. As a result, different electrical lengths are produced for the BLC, leading to a differential phase shift at the output ports. To determine the differential phase shift based on the state of the PIN switches, we assume the input is port 1. The physical lengths of the horizontal and vertical arms of the BLC vary by $∆l_{H}$ and $∆l_{V}$, respectively. Their corresponding lengths are L_H_ and L_V_, as illustrated in Figure 6. The total length of the arms, in the case where the PIN diode is OFF, is given as follows:

(8) $$L_{H}^{\prime }=L_{H}+\bar{S_{H}}∆l_{H}$$

(9) $$L_{V}^{\prime }=L_{V}+\bar{S_{V}}∆l_{V}$$

where S_H_ and S_V_ represent the switch state: 0 when the switch is OFF and 1 when it is ON. $\bar{S_{H}}$ and $\bar{S_{V}}$ are the inversion of S_H_ and S_V_, respectively. The phase delays corresponding to the new arm lengths are as follows:

(10) $$\varphi_{H}^{\prime }=\beta L_{H}^{\prime }$$

(11) $$\varphi_{V}^{\prime }=\beta L_{V}^{\prime }$$

To achieve the maximum phase delay difference between the output ports (port 2 and port 4), let us assume that the switch controlling the current directed to port 2 is ON while both switches associated with port 4 are OFF. Therefore, the phase difference is as follows:

(12) $$\begin{array}{cc} ∠S_{41}-∠S_{21} & =(\varphi_{V}^{\prime }+\varphi_{H}^{\prime })-\varphi_{H})\\ & =\left[\beta \left(L_{v}+\bar{S_{v}}∆l_{V}\right)+\beta \left(L_{H}+\bar{S_{H}}∆l_{H}\right)\right]-\beta L_{H}\\ & =\beta (L_{v}+\bar{S_{v}}∆l_{v}+\bar{S_{H}}∆l_{H}) \end{array}$$

For the same input port, if the switches on the parallel arms are toggled to reverse states, the equation can be written as follows:

(13) $$∠S_{41}-∠S_{21}=\beta (L_{v}-\bar{S_{v}}∆l_{v}-\bar{S_{H}}∆l_{H})$$

However, the general expression for the phase difference between the output ports is the following:

(14) $$∠S_{41}-∠S_{21}=\beta (L_{v}\mp \bar{S_{v}}∆l_{v}\mp \bar{S_{H}}∆l_{H})$$

Therefore, based on the switch states of the arms, differential phase shifts can be achieved. It is worth mentioning that for the same switch states, if the feed is from port 3, then the phase shift relation is written as

(15) $$∠S_{23}-∠S_{43}=\beta (L_{v}\pm \bar{S_{v}}∆l_{v}\pm \bar{S_{H}}∆l_{H}).$$

Thus, when ON/OFF switching is performed on opposite BLC arms (i.e., $\bar{S_{H}}$ and $\bar{S_{V}}$), terms appear or disappear from Equations (14) and (15). Consequently, differential phases are added or removed from the basic phase difference of 90°. It is worth noting that, in addition to achieving a differential phase shift for other cases of switch states, e.g., both $\bar{S_{H}}$ or both $\bar{S_{V}}$ being either 1 or 0, the phase difference remains constant at 90° but occurs at different phase angles. Specifically, ∠S_21_ and ∠S_41_ shift upward or downward by approximately the same amount, and the same applies to ∠S_23_ and ∠S_43_. This scenario becomes particularly useful when employing the proposed design in constructing a Butler matrix, as it can provide the maximum number of differential phases when connected sequentially, as is the case with a BM.

To validate the preceding analysis and confirm the accuracy of Equations (14) and (15), the scattering parameters of the proposed BLC, as shown in Figure 6, are examined. Additionally, the phase differences at the output ports (∠S_21_ and ∠S_41_), as well as at the output ports (∠S_23_ and ∠S_43_), are analyzed under different switch states and compared with the measurements, as presented in the next section.

## 3. Variable Phase BLC Circuit Implementation and Testing

**Figure 8:**
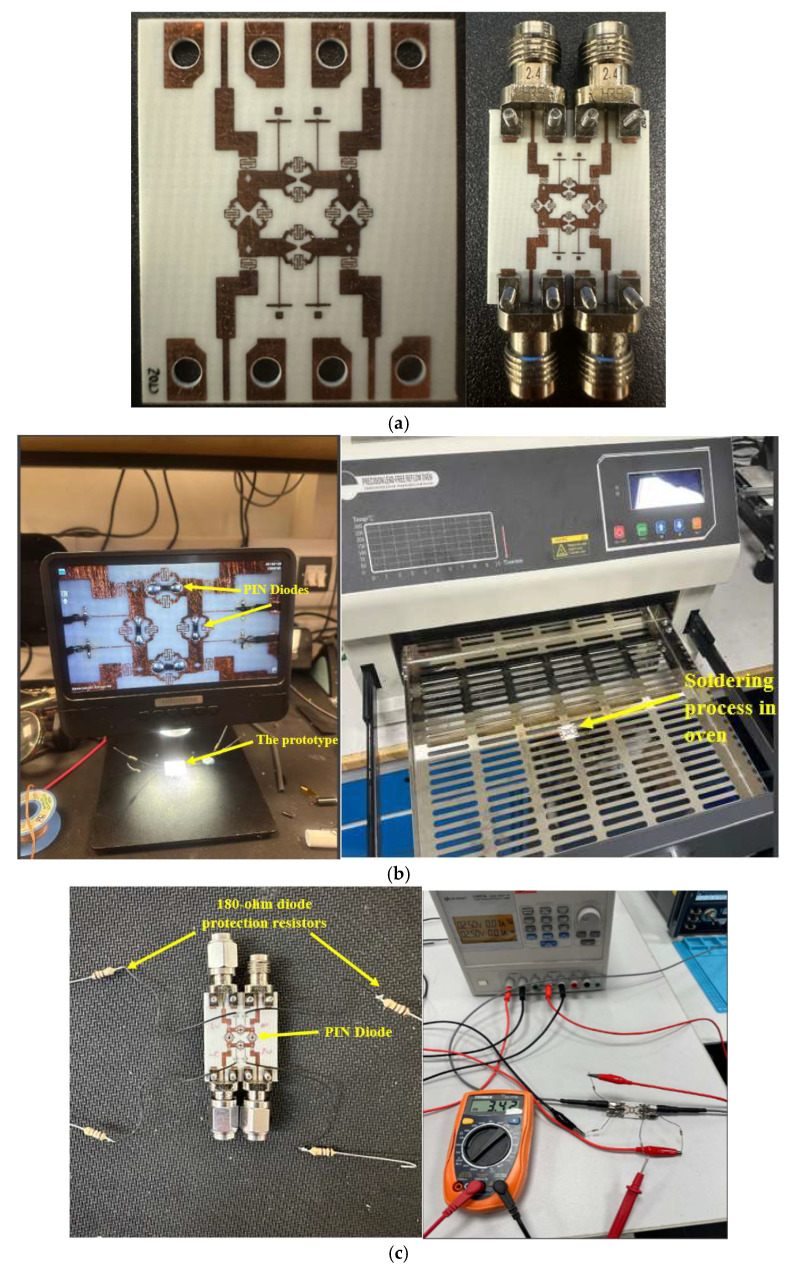
Proposed variable phase shift BLC fabrication process: (**a**) BLC prototype, (**b**) PIN diode integration, (**c**) PIN diode switch biasing test.

To validate the proposed concept and theoretical analysis, the design depicted in Figure 8 was fabricated by JD photodata [32] using an RO4003C Rogers substrate with a thickness of 0.406 mm. A photograph of the fabricated variable phase BLC prototype, implemented with a differential delay transmission line, is presented in Figure 8a. The integration of four PIN diodes was carried out with high precision, with the soldering process performed under a microscope due to the extremely small size of the diodes, which are nearly invisible to the naked eye. Solder paste was used to secure the diodes, and they were subsequently placed in an oven to dry the paste, as illustrated in Figure 8b. Following this, the resistance values connected to the diodes were carefully selected. According to the diode’s datasheet, the maximum tolerable current is 10 mA. To prevent diode burnout, two 180-ohm resistors were connected around each diode. When a voltage of 2.5 V was applied, the current flowing through the diodes was measured as 3.42 mA, as depicted in Figure 8c.

Measurements were conducted utilizing an N5245B vector network analyzer (VNA) equipped with 50-ohm 2.4 mm SMA connectors. The primary performance metrics of the proposed hybrid coupler, including return loss, transmission and coupling coefficients, and phase difference, were evaluated under various configurations of the PIN diode switch states. The four PIN diodes theoretically allow for 16 possible configurations (2^n^), where n denotes the number of diodes. However, not all states were considered in the measurements due to circuit symmetry and practical limitations in implementing individual ON/OFF states for each diode, arising from constraints in the biasing process. Additionally, in certain switch configurations—specifically when both parallel switches are either ON or OFF—the output port phase angles (∠S_21_, ∠S_41_, ∠S_23_, and ∠S_43_) exhibit a uniform shift, maintaining nearly the same angular phase difference. Despite this phase shift, the relative phase difference remains unchanged, indicating that no beam steering occurs when an antenna is connected.

**Figure 9:**
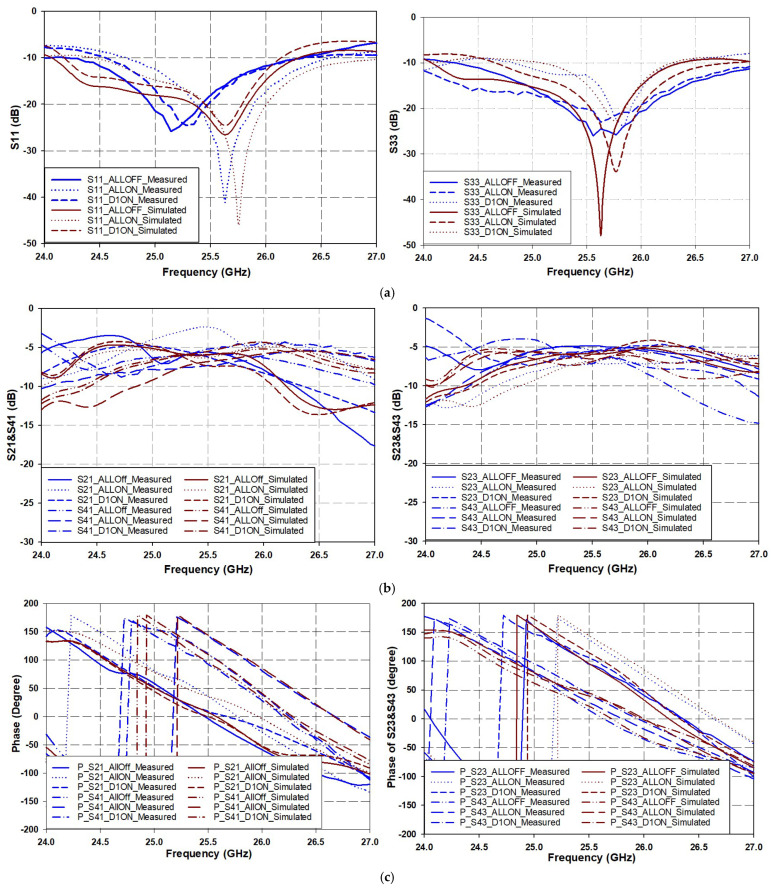
Comparison between simulated and measured scattering parameters of proposed variable phase shift BLC when input is from port 1 (left column) and port 3 (right column). (**a**) Return loss (S_11_ and S_33_), (**b**) transmission and coupling coefficients (S_21_ and S_41_, as well as S_23_ and S_43_), and (**c**) phases of transmission and coupling coefficients (∠S_21_ and ∠S_41_, as well as ∠S_23_ and ∠S_43_).

To avoid excessive curves for multiple switching states, which could make it difficult to observe phase variations, only three cases were considered: those that result in a clear beam steering effect. These cases are ALL ON, ALL OFF, and D1 ON. In this representation, logic ‘1’ denotes the ON state, while logic ‘0’ indicates the OFF state for the diodes D1, D2, D3, and D4. Figure 9a illustrates the comparison between simulated and measured data across the frequency range of 24 GHz to 27 GHz, with a target resonant frequency of 25.5 GHz. The results demonstrate that the proposed BLC achieves excellent matching performance within this frequency band across the selected switch states. Figure 9a indicates that despite a slight shift in the resonant frequency and bandwidth across different diode states, the proposed design exhibits excellent impedance matching at the resonant frequency. The overlapping bandwidth extends from 24.5 GHz to 26.5 GHz for S_11_ and S_33_, except for the D1 ON state, where it starts at 25 GHz.

Figure 9b illustrates the transmission and coupling coefficients, S_21_ and S_41_, respectively, for input port 1, as well as S_23_ and S_42_ for input port 3. It can be observed that the delivered power at the output ports is evenly distributed between them at the resonant frequency, with a bandwidth of 1 GHz, ranging from 25 to 26 GHz, for different switch states.

Referring to Figure 9c, which illustrates the phase difference at the output ports, specifically the phase difference between ∠S_21_ and ∠S_41_, it can be observed that the phase varies with different switch states due to changes in time delay along the current path between the input and output. It is also noteworthy that the phase difference between ∠S_41_ and ∠S_21_ is greater than that between ∠S_23_ and ∠S_43_ for the same switch configuration. This discrepancy arises because the currents flowing from different input ports to the output ports traverse distinct paths depending on the excitation source, thereby validating Equations (14) and (15). The details of the scattering parameters for the proposed design, as illustrated in Figure 9 for different diode states, are presented in Table 3.

From Table 3, it can be observed that the average operating bandwidth is approximately 2.5 GHz. Additionally, the transmission coefficients range from −5 dB to −6 dB, which is expected due to losses of about 2 to 3 dB caused by the forward resistance of the PIN diodes and the coupling loss in the IDC. However, it is noteworthy that the power is evenly distributed among the output ports.

The phase difference at the output ports varies between 62° and 109° depending on the diodes’ states. Furthermore, a variable phase difference is generated in the case of diode asymmetry when the PIN diodes on different branches are in the “ON/OFF” state. In contrast, in the case of symmetry, when the PIN diodes are in the “ON/OFF” state, the phase difference remains relatively constant but occurs at different phase angles.

Table 4 presents a comparison between the proposed design and previous works. The comparison reveals that the main advantage of the current design lies in the fact that it does not require an additional phase shifter circuit to be cascaded with the Butler matrix (BLC). Instead, the phase shifter is creatively integrated within the BLC itself. As a result, the proposed design fulfills the functions of both a conventional Butler matrix and a phase shifter simultaneously.

Moreover, the design can generate both low step and large step phase differences, in addition to offering a wide range of phase variation. Finally, the design operates at millimeter-wave (mmWave) frequencies, which poses a significant challenge due to the inherent difficulty of achieving and implementing phase reconfiguration at such high frequencies. This process typically requires substantial effort and exceptional precision.

## 4. Two-Element Phased Array Antennas

**Figure 10:**
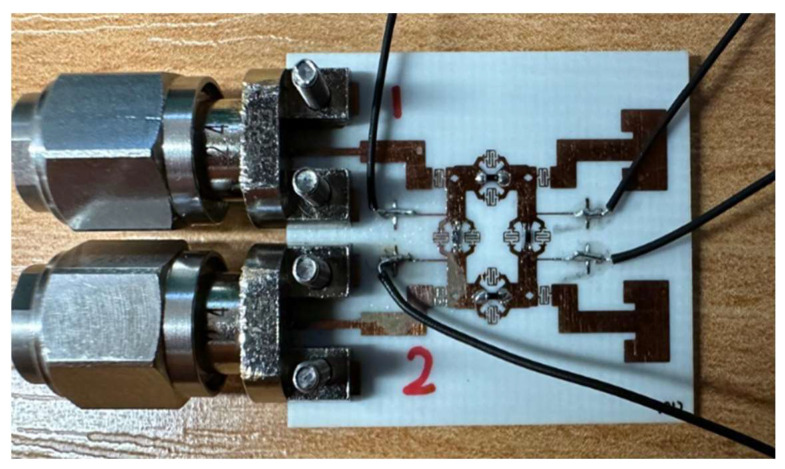
The prototype of the variable phase shift BLC with a two-element antenna.

**Figure 11:**
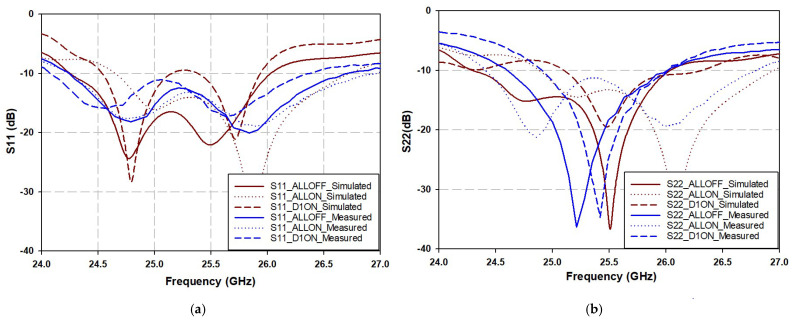
Simulated and measured return loss parameters of proposed two-element variable phase shift BLC antenna array; (**a**) S_11_, (**b**) S_22_.

To verify the ability of the proposed circuit to form and steer the radiation beam in various PIN diode switch modes, it was connected to two conventional microstrip antennas, which were fabricated and tested, as shown in Figure 10. The simulated and measured reflection coefficients for both inputs S_11_ and S_22_ of possible PIN diode switch states are shown in Figure 11. As mentioned earlier, certain diode states produce equal phase differences despite having different phase delays. Consequently, their effect might not appear on beam steering, as the latter is influenced by the phase difference at the output ports rather than the phase delay values themselves. Therefore, distinct cases with varying phase differences were selected, namely 1111, 0000, and 1000. The details of the reflection coefficients for both inputs, S_11_ and S_22_, for different diode states are shown in Table 5.

**Figure 12:**
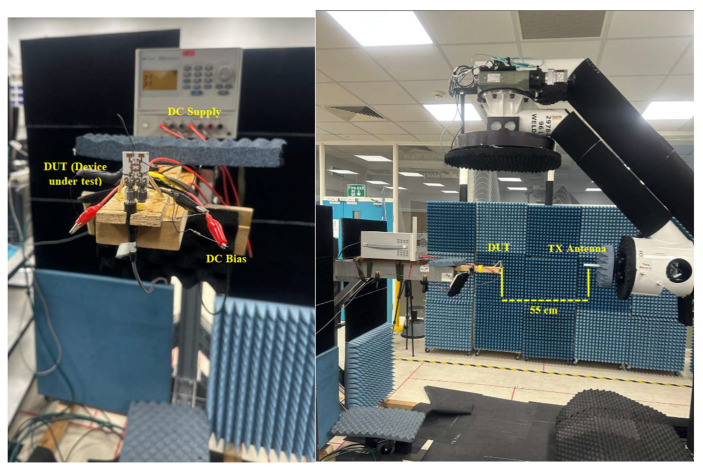
Radiation pattern measurement setup.

The radiation pattern was measured using the measurement setup of Figure 12. The NSI-MI Technologies system was utilized, while the N5245B vector network analyzer (VNA) was used to excite the design. The proposed design was placed 54.9 cm from the reference transmitter antenna to measure its radiation pattern. The DC power supply for biasing the PIN diode was placed behind the antenna, with crocodile-head wires used to excite the diodes, as shown in Figure 12.

**Figure 13:**
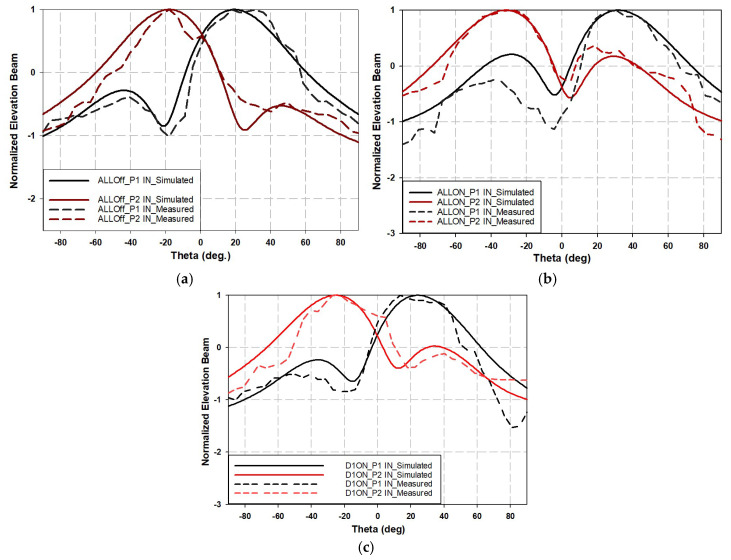
Elevation plane radiation pattern of proposed variable phase shift array antenna for different diode switch states: (**a**) All_OFF, (**b**) ALL_ON, and (**c**) D1_ON.

The normalized elevation plane of the radiation pattern for the three distinct diode states is shown in Figure 13, from which it can be observed that the main beam gradually steers for the same input port, whether it is port 1 (P1-IN) or port 2 (P2-IN). The details of the main beam direction corresponding to each input port and the three distinct cases are provided in Table 6.

It is worth mentioning that the proposed design becomes significantly more important when utilized in larger circuits, such as in the case of the Butler matrix, where four BLCs are arranged sequentially. By incorporating the proposed design into a Butler matrix, more distinctive states and a greater variety of phase differences can be achieved compared to using a single BLC. This will enhance the system’s ability to steer the radiation pattern more gradually and over a wider range, making it highly suitable for 5G mmWave systems.

## 5. Conclusions

A novel variable phase shift BLC for millimeter-wave applications was proposed. The design incorporates an innovative delay line phase shifter structure within the BLC arms. The proposed methodology was formulated through analytical equations and thoroughly investigated through simulation, fabrication, and testing. The measured and simulated results exhibit strong agreement, validating the effectiveness of the design.

The proposed variable BLC enables both fine stepwise phase shifts of approximately 10° and larger stepwise phase variations, depending on the input ports. To verify its feasibility for radiation beam steering, a two-element array antenna was integrated with the variable phase shifter. The results demonstrate the capability of steering the radiation beam from −27° to 27° in the elevation plane.

Furthermore, the significance of the proposed design is amplified when applied to larger circuit configurations, such as a Butler matrix, where four BLCs are arranged sequentially. By incorporating the proposed BLC design into a Butler matrix, a greater number of distinct phase states and a wider range of phase differences can be achieved compared to a conventional single BLC. This enhances the system’s ability to steer the radiation pattern more smoothly and over a broader angular range, making it highly suitable for 5G millimeter-wave communication systems.

## Figures and Tables

**Table 1:** Dimensions of proposed variable phase shift BLC.

Parameter	Dimension in mm	Parameter	Dimension in mm
L_F_	$3\lambda_{g}/2$	$∆l_{v}$	0.9
W_F_	1.21	LS	2.998
L_H_	4.914	L_S2_	1.66
W_H_	1.166	W_CB_	0.25
L_V_	3.1	L_OS_	0.756
W_V_	1.384	L_U_	1.4835
$∆l_{H}$	1.025	S	0.0855

**Table 2:** Details of various diode configurations based on applied DC voltage.

DC Supply Voltages (V)				Diode States			
DC_S1	DC_S2	DC_S3	DC_S4	D1	D2	D3	D4
0	0	0	0	OFF	OFF	OFF	OFF
2.5	0	0	0	ON	OFF	ON	OFF
0	0	0	2.5	OFF	ON	OFF	ON
2.5	0	0	2.5	ON	ON	ON	ON
0	0	−2.5	0	ON	OFF	OFF	ON
0	−2.5	0	0	OFF	ON	ON	OFF
2.5	2.5	0	0	ON	OFF	OFF	OFF
2.5	0	2.5	0	OFF	OFF	ON	OFF

**Table 3:** The details of the scattering parameters of the proposed variable phase shift BLC for various diode states at 25.5 GHz.

**PIN Diode Switch States**				**Input Port 1**											
**D1**	**D2**	**D3**	**D4**	**RL, −10 BW (GHz)**		**S21 (dB)**		**S41 (dB)**		**∠** **S21**		**∠** **S41**		∆θ **$\left(\left|∠S21-∠S41\right|\right)$**	
				**Meas.**	**Sim.**	**Meas.**	**Sim.**	**Meas.**	**Sim.**	**Meas.**	**Sim.**	**Meas.**	**Sim.**	**Meas.**	**Sim.**
0	0	0	0	24–26.3 (2.3)	24–26.3 (2.3)	−5.6	−5.9	−5.8	−5.8	−2.5	7.5	93.9	96.3	98.8	88.8
1	1	1	1	24.7–26.5 (1.8)	24.4–27 (2.6)	−2.47	−6.2	−6.7	−6.7	37.3	36.9	143.7	144.6	105.7	107.7
1	0	0	0	24.4–26.3 (1.9)	24.1–26.1 (2)	−6.4	−6.8	−5.1	−5.4	10.2	0.5	112.3	110.3	102	109.8
**PIN Diode Switch States**				**Input Port 3**											
**D1**	**D2**	**D3**	**D4**	**RL, −10 BW (GHz)**		**S21 (dB)**		**S41 (dB)**		**∠** **S21**		**∠** **S41**		$∆\theta \;(\left|∠S21-∠S41\right|)$	
				**Meas.**	**Sim.**	**Meas.**	**Sim.**	**Meas.**	**Sim.**	**Meas.**	**Sim.**	**Meas.**	**Sim.**	**Meas.**	**Sim.**
0	0	0	0	24.2–27 (2.8)	24.06–26.3 (2.24)	−4.8	−5.2	−5.3	−5.6	95.4	96.2	16.2	22.3	79.2	73.9
1	1	1	1	24–27 (3)	24.6–26.9 (2.3)	−6.5	−6.3	−5.9	−6.3	138.9	144.6	29.6	40	109.3	104.6
1	0	0	0	24.8–26.3 (1.5)	25–26.3 (1.3)	−5.5	−5.4	−6.6	−6.5	106.8	115	43.9	45.3	62.9	69.7

**Table 4:** A comparison between the proposed work and the existing literature.

Reference	Operating Frequency (GHz)	Return Loss dB	Isolation dB	Need for External Phase Shift Circuit	Phase Shift Step Size	Phase Difference Range
[8]	2	20	-	Yes	~10°	151°–(−56°)
[9]	2.4	20	20	Yes	Continues	360°
[10]	2.4	20	20	Yes	22.5°	−90°–(+90°)
[11]	2.4	15	15	Yes	60°~90°	180–(−135°)
[16]	3.5	10	17	Yes	Continues	52°~120°
[19]	1	10	12	No	Continues	45°~135°
This Work	25.5	10	15	No	70°~110°	−3°~145°

**Table 5:** Return loss parameters of proposed variable phase shift array antenna for various diode states.

Diode States				Results		
D1	D2	D3	D4		S11 (GHz) Simulated	S11 (GHz) Measured
0	0	0	0	Feeding from Port 1	24.2–26	24.2–26.7
1	1	1	1		24.6–26.7	24.2–26.9
1	0	0	0		24.5–25.9	24.1–26.3
					S22 (GHz) Simulated	S22 (GHz) Measured
0	0	0	0	Feeding from Port 2	24.2–26	24.6–26
1	1	1	1		24.8–27	24.4–26.8
1	0	0	0		25–26.3	24.8–26

**Table 6:** Main beam details of elevation plane for variable phase shift array antenna under different diode switch states.

Input Port	Diode’s State	Main Beam Direction Angle (deg)		Main Beam AMPLITUDE (dB)	
		Simulated	Measured	Simulated	Measured
1	ALL_OFF	19	18	7.07	5.56
2	ALL_OFF	−18	−18	7.09	6.8
1	ALL_ON	32	27	6.9	6.03
2	ALL_ON	−32	−27	6.9	6.6
1	D1_ON	24	21	6.85	5.9
2	D1_ON	−25	−27	6.93	6.3

## Data Availability

Data are contained within the article.
